# The Screening of Hepatitis B and Hepatitis C Virus Infection among HIV-Infected Inpatients and Evaluation of Correlated Characteristics in a General Hospital in Shenyang, Liaoning, China

**DOI:** 10.3390/jcm11226620

**Published:** 2022-11-08

**Authors:** Chengbo Li, Ying Zhou, Yu Wang, Sheng Liu, Wen Wang, Xu Lu, Cuiming Sun, Pei Liu, Ying Wen

**Affiliations:** Department of Infectious Diseases, The First Affiliated Hospital of China Medical University, Shenyang 110001, China

**Keywords:** HIV, HBV, HCV, diagnostic markers, assessment, Shenyang

## Abstract

**Background**: In this study, we surveyed the prevalence of hepatitis B virus (HBV) surface antigen (HBsAg) and hepatitis C virus (HCV) antibody (anti-HCV) among inpatients with human immunodeficiency virus (HIV) infection and analyzed the correlated factors. **Methods**: We conducted a retrospective data collection of the HIV-infected inpatients in our hospital from January 2010 to December 2020. We utilized multivariate logistic regression to identify the correlated factors. **Results**: The proportion of patients screened for HBsAg was 81.8%, which increased from 66.7% in 2010 to 85.7% in 2020. The proportion of patients with anti-HCV screening was 73.9%, which increased from 58.3% in 2010 to 86.7% in 2020. The prevalence of HBsAg positivity was 10.9%, which decreased from 15.0% in the period of 2010–2015 to 9.0% during 2016–2020. Positive anti-HCV was identified in 4.1% of cases. Compared to 4.8% in the period of 2010–2015, there was a similar prevalence of anti-HCV at 3.1% during 2016–2020. Among the HBsAg-positive cases, HBV deoxyribonucleic acid was screened in 70.8% of cases. Among the anti-HCV positive cases, HCV ribonucleic acid (RNA) was screened in 90% of cases. Albumin < 30 g/L, thrombocytopenia and aspartate aminotransferase (AST) > 40 U/L were associated with HBsAg positivity. AST > 40 U/L and higher CD4-positive T lymphocyte counts were associated with HIV/HCV coinfection. **Conclusions**: The routine screening for both HBV and HCV among HIV-positive inpatients has been greatly improved in the past decade. However, screening for the complete HBV serological markers in HIV-positive inpatients and HCV genotyping among HCV RNA-positive cases leaves much to be desired. A concerted effort should be made to improve HBV vaccine compliance in the HIV-positive population and provide direct-acting antiviral therapies to HCV RNA-positive patients.

## 1. Introduction

In the past 30 years, China has successfully reduced the hepatitis B virus (HBV) endemicity from high to intermediate through HBV vaccination. However, human immunodeficiency virus (HIV)/HBV coinfection remains an urgent concern, because most of the current HIV-positive individuals were predominantly born before the time of routine HBV immunization in China. Compared to an HBV infection chronicity rate of less than 5% among HIV-negative adults, there was a more than 10% chronicity among HIV-positive adults [1,2]. Spontaneous acute hepatitis C virus (HCV) resolution was observed in only 14.4–23% of infections in HIV-positive patients [3,4]. Patients with HBV or HCV coinfection were susceptible to liver damage [5,6]. Compared with HIV-mono-infected patients, HBV- or HCV-coinfected patients had an accelerated progression of cirrhosis and higher liver-related death and all-cause death rates [7,8]. A poorer increase in the CD4-positive T lymphocyte (CD4+T) cell count after antiretroviral therapy (ART) was found among HBV/HIV-coinfected patients [9]. Among the HIV seropositive population, the prevalence of HBV or HCV infection was higher than in the HIV-negative population [10,11]. The percentage of HBV coinfection was 20–30% in sub-Saharan Africa and Asia, while it was only 5–10% in North America, Europe and Australia [12]. The HBV co-infection rate was higher in southern China (14.18%) and western China (10.73%), while it was lower in northern China (6.36%) [13]. The prevalence of the hepatitis B virus surface antigen (HBsAg) was higher among individuals aged ≥40 years than individuals aged ≤40 years [14]. There was a moderate HBV coinfection decrease in China in 2015 compared to 2007 [15]. In recent years, a distinct decrease in the hepatitis C virus antibody (anti-HCV) in the HIV-positive population was observed [14,15]. Intravenous drug users (IVDUs) and men who have sex with men (MSM) had a high prevalence of HCV/HIV coinfection [4,16]. Among HIV-positive individuals with African ancestry in the United Kingdom, the prevalence of anti-HCV and HCV ribonucleic acid (RNA) were 1.3% and 0.17%, respectively [17]. The prevalence of HCV/HIV coinfection was 29.6% in Nepal [16]. Importantly, the practice of HBsAg testing among the HIV-positive population showed notable regional differences, with only 0.7% in Kenya compared to 96.0% in South Africa [18]. Although HBV and HCV are routinely tested for when starting ART in China, they are not routinely tested for among HIV-positive inpatients. Among HIV/HBV-coinfected patients undergoing ART, the seroconversion and reappearance of HBsAg or hepatitis B e-antigen (HBeAg) were frequently observed [19,20]. The reactivation incidence of a former HBV infection [21] and the HBV incidence were observed among followed-up individuals receiving HIV care [22]. The recently acquired HCV infection (RAHC) incidence was also found among MSM with HIV [23,24]. The routine examination for HCV infection among inpatients was recommended [25]. In order to achieve the global strategy aiming to eliminate HBV and HCV by 2030 [26], we conducted a retrospective study of the screening for HBV and HCV infection among HIV-positive inpatients and evaluated the correlated characteristics.

## 2. Methods

### 2.1. Study Population

We retrospectively collected the records of HIV-positive inpatients at the First Affiliated Hospital of China Medical University (a tertiary hospital with 3933 beds in Shenyang, Liaoning, China) from 1 January 2010 to 31 December 2020. The Clinical Research Ethics Committee of China Medical University approved this study. 

### 2.2. Study Design

Clinical and laboratory data were acquired from the cases’ medical records, which were implemented by three authors simultaneously. We analyzed the trend of the screening rates for HBsAg and anti-HCV among the inpatients. We also described the distribution modes of the HBV serological markers among the cases with complete serological results. We reported the data of HBV deoxyribonucleic acid (DNA) detection for the HBsAg-positive samples, HCV RNA detection among the anti-HCV positive cases, and HCV genotyping among the cases with positive HCV RNA. We analyzed the factors that might be categorical variables of HBsAg and HCV/HIV co-infection, including the following: age; sex; education level; local residence in Shenyang (or not); body mass index (BMI); World Health Organization (WHO) HIV clinical stage classification; death during hospitalization (or not); ART status before admission; CD4+T cell count; hemoglobin (HB); platelet (PLT) count; glutamic alanine transaminase (ALT); aspartate aminotransferase (AST); alkaline phosphatase (ALP); gamma-glutamyl transferase (GGT); total bilirubin (TBIL); serum albumin (ALB); serum creatinine (Scr); prothrombin time (PT); and serum sodium concentration. 

### 2.3. Definitions

The complete HBV serological markers consisted of HBsAg, hepatitis B surface antibody (HBsAb), HBeAg, hepatitis B e antibody (HBeAb), and hepatitis B core antibody (HBcAb), which were quantified by a chemiluminescence immunoassay (CLIA) using an “Alinity” instrument (Abbott Laboratories, Lake Forest, IL, USA). The isolated HBsAb was defined as positive HBsAb with negative HBsAg, HBeAg, HBeAb, and HBcAb. The isolated HBcAb was defined as positive HBcAb with negative HBsAg and HBsAb. HCV coinfection was defined as HIV positivity in individuals with both positive anti-HCV and positive HCV RNA. Thrombocytopenia was defined as a platelet count of less than 100,000/μL. Abdominal ultrasound examination or computed tomography scans were the diagnostic procedures used to assess liver cirrhosis or fatty liver disease. Alcohol consumption was defined as patients who drank more than 40 g (20 g for female patients) of alcohol per day for more than 5 years. 

### 2.4. Statistical Analysis

The data were illustrated as numbers and percentages. We compared the conditions between the HBsAg-positive patients and HBsAg-negative patients and between the HIV/HCV-coinfected patients and HIV-mono-infected patients, respectively. The proportions of the categorical variables were compared using the chi-square test or Fisher’s exact test. Univariate and multivariate logistic regression models were employed to assess the factors associated with HIV/HBV coinfection and HIV/HCV coinfection, respectively. The odds ratios (OR), adjusted odds ratios (AOR), and 95% confidence intervals (CI) were displayed. Variables with *p* < 0.10 in the univariate analysis were included in the multivariate logistic models. Only variables with *p* < 0.05 were retained in the final multivariate models. The data were analyzed using the SPSS software for Windows version 22.0 (Chicago, IL, USA).

## 3. Results

### 3.1. The Prevalence of Screening for HBsAg and Anti-HCV among Inpatients with HIV Infection

In total, 1002 hospitalized HIV-infected cases were enrolled in this study, including 916 men (91.4%) and 86 women (8.6%) (Supplementary Table S1). There were 489 (48.8%) cases representing Shenyang local residents and 513 (51.2%) patients from different cities around Shenyang. The median age was 39 years (range: 8–86 years). HIV was sexually transmitted in 881 patients (88.0%), and 541 patients (54.0%) were MSM. The causes of hospital admission in 662 cases (66.1%) were AIDS-related illnesses (at WHO clinical stages III–IV). There were 766 cases (76.4%) whose CD4+T count was <200 cells/µL. There were 325 patients (32.4%) who received ART prior to admission. A total of 248 patients had underlying medical conditions, including hyperlipemia (146 patients), hypertension (62 patients), and diabetes (57 patients). There were 62 cases with alcohol consumption history and 81 cases with fatty livers. All HIV-positive inpatients were Chinese, with Han nationality accounting for 84.5% and other ethnic groups accounting for 15.5%. The HIV inpatients came from our own HIV care clinic and different cities around Shenyang. There were 51 cases who died during hospitalization. Here, we provide a table (Supplementary Table S2) comparing all the data between the sex groups. The results showed that there was no difference in the positivity for HBsAg, the positivity for anti-HCV, and hospitalization mortality between the male and female HIV-positive inpatients. 

**Figure 1:**
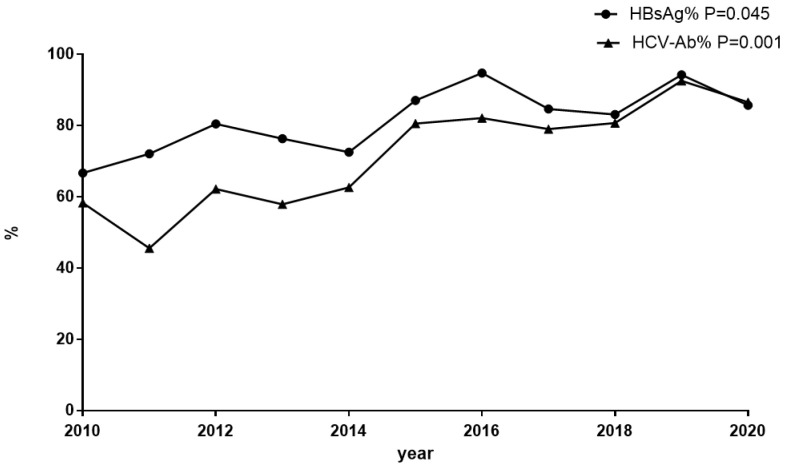
Trend analysis based on a linear-by-linear association test for the screening proportions of HBsAg and anti-HCV. The screening proportions of HBsAg and anti-HCV showed an ascending trend among the HIV-positive inpatients in the past several years. HBsAg: the screening proportion of HBsAg among HIV-positive inpatients. HCV-Ab: the screening proportion of anti-HCV among HIV-positive in patients.

**Figure 2:**
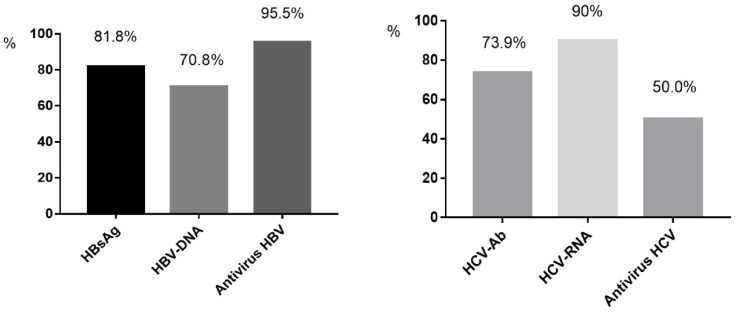
The proportion of HIV-positive inpatients accepting screening and anti-virus treatment. HBsAg: the screening proportion of HBsAg among HIV-positive inpatients. HCV-Ab: the screening proportion of anti-HCV among HIV-positive in patients. HBV-DNA: the screening proportion of HBV-DNA among HBV/HIV co- infected inpatients. HCV-RNA: the screening proportion of HCV-RNA among anti-HCV- positive inpatients with HIV infection. Antivirus HBV: the proportion of anti- HBV treatment among HBV/HIV co- infected patients. Antivirus HCV: the proportion of anti-HCV treatment among HCV/HIV co-infected inpatients.

The proportion of patients screened for HBsAg was 81.8% (820/1002), which increased from 66.7% in 2010 to 85.7% in 2020 overall (*p* = 0.045) (Figure 1). Among the 820 individuals tested, 89 (10.9%) cases were HBsAg-positive, and 37.1% (33/89) of these patients were aware of their HBV-infected condition before hospitalization. The HBsAg prevalence was 12.5% (52/415) in the 30–50-year age group and 11.6% (20/172) in the <30-year age group, while it was only 7.3% (17/233) in the >50-year age group (*p* > 0.05). For the HBsAg-positive samples, HBV DNA was detected in 70.8% (63/89) of cases, including 46 cases with HBV DNA beyond 10,000 IU/mL, 13 cases with between 100 IU/mL and 10,000 IU/mL, and 4 cases with under 100 IU/mL. Compared to the 15.0% prevalence of HBsAg in the period of 2010–2015, there was a moderate decrease to 9.0% during the 2016–2020 period (*p* = 0.01). At discharge, 95.5% (85/89) of the HBsAg-positive cases received ART containing tenofovir disoproxil fumarate (TDF) or tenofovir alafenamide (TAF) (Figure 2). 

The percentage of patients screened for anti-HCV was 73.9% (740/1002), which increased from 58.3% in 2010 to 86.6% in 2020 overall (*p* = 0.001) (Figure 1). Positive anti-HCV was found in 4.1% (30/740) of cases. The positive anti-HCV prevalence was 5.5% (11/201) in the >50-year age group and 4.4% (16/362) in the 30–50-year age group, while it was only 1.9% (3/161) in the <30-year age group (*p* > 0.05). Compared to 4.8% in the period of 2010–2015, there was a similar 3.1% prevalence of anti-HCV during 2016–2020 (*p* > 0.05). Of the anti-HCV-positive cases, HCV RNA was detected in 90.0% (27/30), and nine of these tested negative for HCV RNA. Among 18 cases with positive HCV RNA (HIV/HCV coinfection), 4 cases were tested for genotypes. At discharge, 50% (9/18) of the HCV RNA-positive cases received anti-HCV therapy (Figure 2). No patients had HCV/HBV/HIV tri-infection.

### 3.2. Distribution of HBV Serological Markers among Cases with Complete Serological Results

There were 487 cases with complete HBV serological markers, and 47 cases (9.7%) were HBsAg-positive. Among the 47 HBsAg-positive cases, 48.9% (23/47) of cases were HBeAg-positive. Isolated HBsAb positivity among the inpatients with HIV infection was 26.5% (129/487). The highest isolated HBsAb prevalence was 40.6% (43/106) in the <30-year age group. The lowest isolated HBsAb prevalence was 16.2% (24/148) in the >50-year age group. Among the 440 HBsAg-negative cases, 43.9% (193/440) of cases were HBcAb-positive, including 92 cases with isolated HBcAb. The rate of negative results for all the HBV serological tests among the inpatients with HIV infection was 24.2% (118/487) (Table 1). 

### 3.3. Clinical Characteristics at Baseline among HIV/HBV-Coinfected Patients

Among the HBsAg-positive participants, 11.2% (10/89) of cases had cirrhosis (4 cases of Child–Pugh class B and 6 cases of Child–Pugh class C). Compared with the patients with HIV monoinfection, the cases with HBV coinfection were more likely to have thrombocytopenia (*p* = 0.001), ALB < 30 g/L (*p* = 0.036), ALT > 50 U/L (*p* = 0.038), AST > 40 U/L (*p* = 0.001), ALP > 100 U/L (*p* = 0.002), and PT > 13.7 S (*p* = 0.003) (Table 2). The multivariate logistic regression analysis showed that thrombocytopenia (*p* = 0.001), ALB < 30g/L (*p* = 0.024), and AST > 40 U/L (*p* = 0.004) were characteristic of the HBsAg-positive cases (Table 3).

### 3.4. Clinical Characteristics at Baseline among HIV/HCV-Coinfected Patients

Among the 18 HIV/HCV-coinfected patients, two cases had cirrhosis of Child–Pugh class A, and 55.6% (10/18) of the patients were aware of their HCV-infected status before hospitalization. Compared with the patients with HIV monoinfection, the patients with HCV coinfection were more likely to present with ALT > 50 U/L (*p* = 0.018), AST > 40 U/L (*p* < 0.022), ALP > 100 U/L (*p* = 0.014), and an age of < 30 years (*p* = 0.047), while they were less likely to have CD4+T counts of <200 cells/µL (*p* = 0.001) (Table 2). The multivariate logistic regression analysis showed that AST > 40 U/L (*p* = 0.013) and higher CD4+T counts (*p* = 0.001) were characteristics of the HCV RNA-positive cases (Table 3).

## 4. Discussion

The screening for both HBV and HCV among HIV-positive inpatients at our hospital has greatly improved in the past decade, but it still falls short of the 2030 target of the diagnosis of viral hepatitis B and C in 90% of cases [26]. As one of the populations most affected by HBV and HCV infection, testing for HBV and HCV in the HIV-positive population was recommended [26]. The practice of HBsAg testing among the HIV population varies widely across countries, remaining below 50% in most countries across sub-Saharan Africa [18]. Although we observed that HBsAg screening has increased considerably over time, the screening for the complete HBV serological markers leaves much room for improvement, which is considered as the optimal HBV screening strategy for China within the next 10 years [27]. 

The HBsAg-positive rate was 10.9% in this study, which is higher than the 6.1–7.43% rate among the general population [28,29]. In this study, no statistically relevant age difference in the HBsAg prevalence was found, whereas the highest HBsAg prevalence was previously reported in patients who were 41–50 years old [28]. Isolated HBsAb positivity in this study was 26.5%, which was higher than the rate of 7.7% in the HIV-positive population [30] and similar to the rate of 27% in the general population [28,31]. 

Considering the high prevalence of occult hepatitis B in HIV-positive patients [32], detecting HBV DNA is necessary, especially for the HBsAg-negative patients with positive HBcAb [33]. There is a higher HBV DNA load and HBeAg percentage among coinfected individuals compared to HBV-mono-infected cases [34]. The prevalence of HBeAg among the HBsAg-positive cases in this study was 48.9%, which was previously reported as ranging from 24.4% to 48.5% in the HIV-positive population [34,35] and higher than the rate of 31.04% in the general population [28]. 

The prevalence of isolated HBcAb can be high in HCV- or HIV-infected patients and other types of immunocompromised patients. Isolated HBcAb positivity rate was 18.9% in this study, which is within the previously reported range of 7% to 40%. Thrombocytopenia, ALB < 30 g/L, and AST > 40 U/L were the correlated characteristics of HBsAg positivity in this study. Higher ALT levels and lower PLT counts were correlated with advanced fibrosis among HIV/HBV-coinfected patients [36]. The PT and international normalized ratios were reported to be significantly high among coinfected individuals [34]. HIV/HBV-coinfected patients are associated with lower CD4+T cell counts [37]. HBV/HIV coinfection, especially in cases with low nadir CD4+T cell counts, is associated with increased liver disease progression and liver-related mortality [38]. 

In this study, we observed that anti-HCV screening has increased considerably over time, a finding analogous to that of a previous report [25]. The rate of anti-HCV positivity was only 4.1% in this study, which is higher than the rate of 0.88–3.0% in the general population [26,29]. Most of the HIV-positive cases in our study were due to sexual transmission and had a lower HCV infection rate than IVDUs and former plasma donors. Screening for anti-HCV alone among HIV-infected patients was not enough. In total, 90% of anti-HCV-positive cases were screened for HCV RNA in this study, while HCV genotyping leaves much to be desired. Moreover, we should also pay attention to the high rate of occult hepatitis C in HIV-positive patients with IVDU [39]. Higher AST levels and higher CD4+T cell counts were found among the HIV/HCV-coinfected inpatients, because the abnormal liver tests were the main cause of hospital admission for a number of HIV/HCV-coinfected patients. An AST >40 IU/L was correlated with HCV co-infection [40]. An age >35 years, alcohol abuse, and a decrease in the CD4 cell count were identified as risk factors for fibrosis progression in HIV/HCV-coinfected patients [41]. Persistently normal aminotransferases in HIV/HCV-coinfected patients undergoing antiretroviral therapy only show minimal liver cirrhosis development [42]. Furthermore, the improvements in anti-HCV and HIV testing among general outpatients and inpatients resulted in the earlier discovery of HIV/HCV-coinfected cases.

The limitations of our study were the small sample size and the lack of follow-up detection. Therefore, the HBV or HCV incidence rates were not obtained during long-term follow-up. Additionally, we only enrolled inpatients at our hospital, which allowed us to collect detailed information from their medical records. This study highlighted the necessity of the frequent monitoring of complete HBV serum markers and HBV DNA among the HIV-positive population. Despite being on ART including lamivudine and tenofovir, HBV viremia was still found in 28.6% of HIV/HBV-coinfected patients [43]. Although the immunogenicity of the HBV vaccination in HIV-infected patients is inferior to that among the HIV-seronegative population, this could be notably improved by a double dose of the HBV vaccine [44]. Among cases with isolated HBcAb, monitoring and vaccination should also be recommended [45,46]. Indeed, poor vaccine compliance was identified among the HIV-infected population, which was associated with a lack of insurance coverage and low educational level [47]. For the anti-HCV-positive cases, HCV RNA and HCV genotyping should be recommended. The Direct acting antiviral agents (DAA) achieved an identical efficacy between patients with HCV monoinfection and patients with HIV/HCV coinfection.

## 5. Conclusions

The viral hepatitis epidemic in HIV-positive populations continues to be a concern. The feasible practice recommendations include the integration of screening for HBV or HCV serological markers with routine physical examinations, professional training of healthcare workers, education of the general population, budgeting of the Chinese health-care system, and expansion of the mechanism of linkage-to-care [48]. HBV vaccination is recommended for people living with HIV for preventing horizontal transmission of HBV. It is crucial to keep TDF or TAF in the anti-HIV regimen among HIV/HBV coinfected cases, even in those with a clinically cured HBV infection. Importantly, optimizing the provision of DAA to HIV/HCV coinfected patients, monitoring for the recurrence and reinfection of HCV, and tracking RAHC incidence may eliminate some of the barriers to achieving HCV eradication.

## Figures and Tables

**Table 1:** The distribution of different modes of HBV serological markers among HIV-positive inpatients.

Modes	Group	*n*	HBsAg+ HBeAg+ HBcAb+ [*n* (%)]	HBsAg+ HBeAg− HBcAb+ [*n* (%)]	HBsAg− HBSAb− HBcAb+ [*n* (%)]	HBsAg− HBSAb+ HBcAb+ [*n* (%)]	HBsAg− HBSAb+ HBcAb− [*n* (%)]	HBsAg− HBSAb− HBcAb− [*n* (%)]
Age	<30	106	6(1.2)	4(0.8)	8(1.6)	3(0.6)	43(8.8)	42(8.6)
	30–50	233	8(1.6)	11(2.3)	51(10.5)	51(10.5)	62(12.7)	50(10.3)
	>50	148	9(1.9)	9(1.9)	33(6.8)	47(9.7)	24(4.9)	26(5.3)
Sex	male	443	22(4.5)	18(3.7)	88(18.1)	88(18.1)	124(25.5)	103(21.2)
	female	44	1(0.2)	6(1.2)	4(0.8)	13(2.7)	5(1.0)	15(3.1)
	total	487	23(4.7)	24(4.9)	92(18.9)	101(20.7)	129(26.5)	118(24.2)

**Table 2:** The baseline clinical features of HBV/HIV- or HCV/HIV-coinfected inpatients.

Characteristics	HBSAg+ (*n*, %)	HBsAg− (*n*, %)	P1	HCV-RNA+ (*n*, %)	HCVAb− (*n*, %)	P2
*n*	89(100.0)	731(100.0)		18(100.0)	710(100.0)	
Age < 30 30–50 >50	20(22.5)	152(20.8)		0(0.0)	158(22.3)	
	52(58.4)	363(49.7)		10(55.6)	363(51.1)	
	17(19.1)	216(29.5)	0.113	8(44.4)	190(26.8)	0.047 *
College education	27(30.3)	213(29.1)	0.814	4(22.2)	216(30.4)	0.606
Sex (male)	82(92.1)	662(90.6)	0.161	16(88.9)	650(91.5)	0.690
Local residents	45(50.6)	341(46.7)	0.485	11(61.1)	332(46.8)	0.228
WHO clinical stage III–IV	60(67.4)	492(67.3)	0.983	9(50)	493(69.4)	0.078
Alcohol consumption history	9(10.1)	45(6.2)	0.161	1(5.6)	48(6.8)	0.587
Fatty liver	7(7.9)	55(7.5)	0.921	1(5.6)	56(7.9)	0.989
Underlying medical conditions	21(23.6)	185(25.3)	0.703	2(11.1)	176(24.8)	0.743
ART prior to admission	32(36.0)	224(30.6)	0.311	7(38.9)	232(32.7)	0.582
HB < 9 g/dL	12(13.5)	68(9.3)	0.209	2(11.1)	73(10.3)	0.909
Thrombocytopenia	16(18.0)	45(6.2)	0.001 *	3(16.7)	53(7.5)	0.148
ALT > 50 U/L	31(34.8)	180(24.6)	0.038 *	9(50)	180(25.4)	0.018 *
AST > 40 U/L	43(48.3)	208(28.5)	0.001 *	10(55.5)	215(30.3)	0.022 *
ALP > 100 U/L	38(42.7)	196(26.8)	0.002 *	10(55.5)	204(28.7)	0.014 *
GGT > 60 U/L	49(55.1)	362(49.5)	0.324	12(66.7)	356(50.1)	0.166
TBIL > 17.1 µmol/L	1(1.1)	17(2.3)	0.728	0(0.0)	18(2.5)	1.000
Serum ALB < 30 g/L	46(51.7)	266(36.4)	0.036 *	3(16.7)	278(39.2)	0.053
PT > 13.7 S	51(57.3)	298(40.8)	0.003 *	7(38.9)	303(42.7)	0.748
Serum Na < 135 µmol/L	29(32.6)	222(30.4)	0.669	6(33.3)	225(31.7)	0.882
Scr > 104 μmol/L	4(4.5)	13(1.8)	0.090	1(5.6)	17(2.4)	0.394
CD4 < 200 /µL	74(83.1)	557(76.2)	0.219	8(44.4)	556(78.3)	0.001 *
BMI < 18	9(10.1)	118(16.1)	0.106	2(11.1)	110(15.5)	0.751
Death	4(4.5)	36(4.9)	0.859	1(5.6)	33(4.6)	0.857

* *p* value < 0.05, statistically significant with the use of the chi-square test or Fisher’s exact test. P1, between HBSAg+ and HBSAg- patients with HIV infection; P2, between HIV/HCV-coinfected and HIV-monoinfected patients. ART, antiretroviral therapy; HB, hemoglobin; Scr, serum creatinine; ALT, alaninetransaminase; AST, aspartate aminotransferase; BMI, body mass index; ALP, alkaline phosphatase; GGT, gamma-glutamyltransferase; TBIL, total bilirubin; DBIL, direct bilirubin; PT, prothrombin time.

**Table 3:** Factors associated with HBV or HCV coinfection in HIV-positive inpatients.

	HIV/HBV-Coinfected				HIV/HCV-Coinfected			
Factors	OR(95%CI)	P1 Value	Adjust OR(95%CI)	P2 Value	OR(95%CI)	P3 Value	Adjust OR(95%CI)	P4 Value
ALT > 50 U/L								
No	1.000				1.000			
Yes	1.636(1.198–3.029)	0.039			2.944(1.151–7.532)	0.024		
AST > 40 U/L								
No	1.000		1.000		1.000			
Yes	2.350(1.505–3.670)	0.043	1.959(1.235–3.109)	0.004	2.878(1.120–7.392)	0.028	3.380(1.292–8.841)	0.013
ALP > 100 U/L								
No	1.000				1.000			
Yes	2.034(1.296–3.192)	0.002			3.100(1.207–7.967)	0.019		
Thrombocytopenia								
No	1.000		1.000					
Yes	3.341(1.798–6.208)	0.001	3.061(1.621–5.780)	0.001				
ALB < 30 g/L								
No	1.000		1.000		1.000			
Yes	1.870(1.202–2.910)	0.006	1.698(1.074–2.685)	0.024	0.311(0.089–1.083)	0.067		
PT > 13.7 S								
No	1.000							
Yes	1.950(1.249–3.044)	0.003						
Scr > 104 μmol/L								
No	1.000							
Yes	1.383(0.388–4.933)	0.090						
WHO clinical stage III–IV								
No					1.000			
Yes					0.440(0.172–1.124)	0.078		
CD4 < 200/µL								
No					1.000			
Yes					0.222(0.086–0.571)	0.002	0.195(0.075–0.510)	0.001

P1, *p*-value in the univariable analysis of HIV/HBV-coinfected patients; P2, *p*-value in the multivariable logistic regression models for HIV/HBV-coinfected patients; P3, *p*-value in the univariable analysis of HIV/HCV-coinfected patients; P4, *p*-value in the multivariable logistic regression models for HIV/HCV-coinfected patients.

## Data Availability

Data are available on request to the authors.

## Supplementary Materials

The following supporting information can be downloaded at: https://www.mdpi.com/article/10.3390/jcm11226620/s1, Table S1: The demographic and clinical features of HIV-positive inpatients; Table S2: Comparison of the baseline clinical features between male inpatients and female inpatients with HIV infection.

## Abbreviations

HBsAg	hepatitis B surface antigen
anti-HCV	hepatitis C virus antibody
HBV	hepatitis B virus
HIV	human immunodeficiency virus
HCV	hepatitis C virus
IVDUs	intravenous drug user
MSM	men who have sex with men
RNA	ribonucleic acid
ART	antiretroviral therapy
HBeAg	hepatitis B e-antigen
RAHC	recently acquired HCV infection
DNA	deoxyribonucleic acid
BMI	body mass index
WHO	World Health Organization
HB	hemoglobin
PLT	platelet
ALT	glutamic alanine transaminase
AST	aspartate aminotransferase
ALP	alkaline phosphatase
GGT,	gamma-glutamyltransferase
TBIL	total bilirubin
ALB	albumin
Scr	serum creatinine
PT	prothrombin time
HBsAb	hepatitis B surface antibody
HBeAb	hepatitis B e antibody
HBcAb	hepatitis B core antibody
CLIA	chemiluminescence immunoassay
OR	odds ratios
AOR	adjusted odds ratios
CI	confidence intervals
OBI	occult hepatitis B
OCI	occult hepatitis C
TDF	tenofovir disoproxil fumarate
TAF	tenofovir alafenamide
DAAs	direct-acting antiviral therapies
