# Supplementary material for: The Screening of Hepatitis B and Hepatitis C Virus Infection among HIV-Infected Inpatients and Evaluation of Correlated Characteristics in a General Hospital in Shenyang, Liaoning, China
Source: J Clin Med. 2022 Nov 8;11(22):6620. doi: 10.3390/jcm11226620 (PMC9692379; doi:10.3390/jcm11226620)
Supplement: Supplementary file 1 [file jcm-11-06620-s001.zip › jcm-1963729-supplementary.pdf]

Supplementary Table S1. The demographic and clinical features of HIV-positive inpatients

Characteristics	No.(%)
n	1002(100.0)
Age <30	212(21.2)
30-50	510(50.9)
>50	280(27.9)
College education	297(29.6)
Sex(male)	916(91.4)
Local residents	489(48.8)
WHO clinical stage III-IV	662(66.1)
Alcohol consumption history	62(6.2)
Fatty liver	81(8.1)
Underlying medical conditions	248(24.8)
HB<9g/dl	120(12.0)
ART prior to admission	325(32.4)
CD4<200 /μL	766(76.4)
BMI<18	155(15.5)
Death	51(5.1)

ART, antiretroviral therapy; HB, hemoglobin;BMI, body mass index;

Supplementary Table S2. Comparison of the baseline clinical features between male inpatients and female inpatients with HIV infection

Characteristics	Female (n=86)	Male (n=916)	P
n=1002	86(8.6)	916(91.4)	
Age <30 [n (%)]	6(7.0)	206(22.5)	
30-50 [n (%)]	38(44.2)	472(51.5)	
>50 [n (%)]	42(48.8)	238(26.0)	0.001*
College eucation [n (%)]	11(12.8)	286(33.2)	0.001*
HBSAg+ [n (%)]	7/66(10.6)	82/754(10.9)	0.161
Anti-HCV+ [n (%)]	5/66(7.6)	25/674(3.7)	0.170
Local residents [n (%)]	37(43.0)	452(49.3)	0.262
WHO clinical stage III-IV [n (%)]	45(52.3)	617(67.4)	0.005*
Alcohol consumption history	2(2.3)	62(6.8)	0.158
Fatty liver	5(5.8)	76(8.3)	0.481
Underlying medical conditions	23(26.7)	225(24.6)	0.654
ART prior to admission [n (%)]	14(16.3)	311(34.0)	0.001*
HB<9g/dl [n (%)]	6(7.0)	114(12.5)	0.135
Thrombocytopenia [n (%)]	7(8.1)	86(9.4)	0.703
ALT>50U/L [n (%)]	12(14.0)	232(25.3)	0.109
AST>40 U/L [n (%)]	13(15.1)	280(30.6)	0.003*
ALP>100 U/L [n (%)]	14(16.2)	279(30.5)	0.006*
GGT>60U/L [n (%)]	18(20.9)	480(52.4)	0.001*
TBIL>17.1 μmol/L[n (%)]	1(1.2)	23(2.5)	0.714
Serum ALB<30g/L [n (%)]	22(25.6)	376(41.0)	0.005*
PT>13.7 S [n (%)]	28(32.6)	394(43.0)	0.060
Serum Na<135μmol/L [n (%)]	11(12.8)	304(33.2)	0.000*
Scr>104 μmol/L [n (%)]	0(0)	21(2.3)	0.247
CD4<200 /μL[n (%)]	59(68.6)	707(77.2)	0.073
BMI<18 [n (%)]	17(19.8)	138(15.1)	0.249
Death [n (%)]	5(5.8)	46(5.0)	0.749

*P value <0.05, statistically significant with the use of chi-square test or Fisher's exact test.

ART, antiretroviral therapy; HB, hemoglobin; Scr, serum creatinine; ALT, alaninetransaminase; AST, aspartate aminotransferase; BMI, body mass index; ALP, alkaline phosphatase; GGT, gamma-glutamyltransferase; TBIL, total bilirubin; DBIL, direct bilirubin; PT, prothrombin time.
